# Model of dynamics of intracellular chloride based on fluorescent imaging

**DOI:** 10.1186/1471-2202-15-S1-P169

**Published:** 2014-07-21

**Authors:** Shiva Ghaani, Jean F Lienard, Susan L Ingram, Alexander G Dimitrov

**Affiliations:** 1 Department of Mathematics, Washington State University, Vancouver, WA, USA; 2 Department of Neurological Surgery, Oregon Health & Science University, Portland, OR, USA

## 

Fluorescent indicators are relatively non-invasive probe that allow the measure of ion concentrations in brain slices and neuron cultures using microscopy imaging. Most fluorescent ion indicators bind selectively with a certain ion, causing a decrease in fluorescence in a process known as quenching. Under steady state conditions, a fluorescence measurement, made at a specific point and time, is directly related to the local ion concentration at the same point and time, typically via the Stern-Volmer relationship. However, this is usually no longer true under the dynamic conditions inside a cell when transmembrane currents are active. While calcium ions (Ca²^+^) dynamics have been well analyzed theoretically [1], chloride ions (Cl^-^) indicators have been experimentally shown to exhibit changes in the timescale of minutes following the transient bathing with a GABA A agonist – leading to the interpretation that Cl^-^ dynamics are very slow [2].

In this follow-up of a theoretical analysis of chloride indicator dynamics [3], we present novel contributions to the understanding of Cl^-^ dynamics through the computational modeling of a recently built genetic membrane-bound fluorescent indicator (mbYFPQS, [4]). This indicator, obtained by adding a palmitoylation sequence to a previously described chloride indicator (YFPQS) to target the protein to the plasma membrane of cultured midbrain neurons, offers the possibility to study the mechanisms of Cl^-^ transmembrane exchanges with an unprecedented resolution. We designed a computational model allowing the study of Cl^-^ based on a detailed multi-compartmental conductance model that includes (a) Cl^-^ pumps as well as (b) the interaction of Cl^-^ with the mbYFPQS indicator as a system of non-linear differential equations derived from biophysical and chemical kinetic theory. To constrain the model parameterizations, we relied on fluorescent measurements of mbYFPQS recorded in rat cultured midbrain cells, and during which the extracellular medium was replaced several times by a muscimol bath followed by a wash solution. Using tools from the optimization theory to fit the model outputs with these experimental data, we were able to find broad plausible boundaries for parameters such as the Cl^-^ leak pump kinetics as well as the forward/backward binding rates with the fluorescent indicator. However, the observed data did not have a sufficient temporal precision to derive precise estimates of these parameters. We also established through a sensitivity analysis on the membrane indicator density that orders of magnitude from 10^-11^ (e.g., *as dense as pumps*) to 10^-18^ M.cm^−2^ (e.g., *as dense as postsynaptic densities*) did not change the best fit parameter ranges nor the overall goodness-of-fit with experimental data, thus validating the widespread assumption that binding dynamics are independent from the indicator concentration in neurons.
